# Intra-Peritoneal Hyperthermia Combining α-Galactosylceramide in the Treatment of Ovarian Cancer

**DOI:** 10.1371/journal.pone.0069336

**Published:** 2013-07-23

**Authors:** Chao-Chih Wu, Yin-Ting Chuang, Yun-Ting Hsu, Jung-Tang Huang, T. -C Wu, Chien-Fu Hung, Yuh-Cheng Yang, Chih-Long Chang

**Affiliations:** 1 Graduate Institute of Mechanical and Electrical Engineering, National Taipei University of Technology, Taipei City, Taiwan; 2 Department of Medicine, Mackay Medical College, New Taipei City, Taiwan; 3 Institute of Biomedical Science, Mackay Medical College, New Taipei City, Taiwan; 4 Department of Obstetrics and Gynecology, Mackay Memorial Hospital, Taipei City, Taiwan; 5 Department of Medical Research, Mackay Memorial Hospital, Taipei City, Taiwan; 6 Department of Pathology, The Johns Hopkins University, Baltimore, Maryland, United States of America; 7 Department of Oncology, The Johns Hopkins University, Baltimore, Maryland, United States of America; 8 Department of Obstetrics and Gynecology, The Johns Hopkins University, Baltimore, Maryland, United States of America; 9 Department of Molecular Microbiology and Immunology, The Johns Hopkins University, Baltimore, Maryland, United States of America; Aaron Diamond AIDS Research Center with the Rockefeller University, United States of America

## Abstract

The purpose of this study was to investigate the anti-tumor effect and potential mechanisms of i.p. hyperthermia in combination with α-galactosylceramide (α-GalCer) for the treatment of ovarian cancer. In this study, immuno-competent tumor models were established using murine ovarian cancer cell lines and treated with i.p. hyperthermia combining α-GalCer. Th1/Th2 cytokine expression profiles in the serum, NK cell cytotoxicity and phagocytic activities of dendritic cells (DCs) were assayed. We also analyzed the number of CD8^+^/IFN-γ^+^ tumor specific cytotoxic T cells, as well as the tumor growth based on depletion of lymphocyte sub-population. Therapeutic effect on those ovarian tumors was monitored by a non-invasive luminescent imaging system. Intra-peritoneal hyperthermia induced significant pro-inflammatory cytokines expression, and sustained the response of NK and DCs induced by α-GalCer treatment. The combination treatment enhanced the cytotoxic T lymphocyte (CTL) immune response in two mouse ovarian cancer models. This novel treatment modality by combination of hyperthermia and glycolipid provides a pronounced anti-tumor immune response and better survival. In conclusion, intra-peritoneal hyperthermia enhanced the pro-inflammatory cytokine secretion and phagocytic activity of DCs stimulated by α-GalCer. The subsequent CTL immune response induced by α-GalCer was further strengthened by combining with i.p. hyperthermia. Both innate and adaptive immunities were involved and resulted in a superior therapeutic effect in treating the ovarian cancer.

## Introduction

Although ovarian cancer accounts for only 3% of all malignancies in women, it is the most lethal gynecologic malignancy worldwide [1]. Due to the absence of specific symptoms or signs and lack of reliable screening modalities in early disease, ovarian cancer is mostly diagnosed at an advanced stage [2]–[4]. Currently, the standard upfront treatment for epithelial ovarian cancer consists of maximal cytoreductive surgery followed by adjuvant chemotherapy with platinum-taxane combination regimen. Although response rates are about 70–80%, the majority of these patients will eventually relapse. Despite the increasing number of chemotherapeutic drugs available for recurrent disease, the course of ovarian cancer is usually characterized by repeated periods of remission and relapse, and in most cases the disease will eventually develop resistance to all chemotherapeutic agents and become incurable [5]. Based on the biologic behavior and spreading pattern of ovarian cancer cells, several i.p. treatment modalities have been advocated to achieve better disease control [6]. Among them, i.p. hyperthermia, especially when combined with chemotherapy, has been shown to improve the therapeutic efficacy for either optimally or sub-optimally debulked ovarian cancer [7], [8]. It has been reported that high temperature could induce direct thermal cytotoxicity and also bring “thermal chemo-sensitization” [9]. It is generally believed that the sensitization results from increased tissue perfusion and tissue penetration of the cytotoxic agents. Although the basic mechanisms of hyperthermia have been generally well accepted, the application of therapeutic hyperthermia in conjunction with chemotherapy to the patients is much more complex [10]. Hyperthermic chemotherapy (42–43°C) has been known to be associated with greater cytotoxicity to cancer cells. However, there are some drawbacks limiting its usage in treating the ovarian cancer, such as potential poor-healing of bowel anastomosis, risk of chemotherapeutic metabolites contamination to the surgical personnel through body fluid or inhalation of evaporating chemotherapy agents [11]. Moreover, it cannot be applied repeatedly without exploration of the peritoneal cavity.

Hyperthermia itself has been shown to exert a series of cellular and molecular effects, especially in eliciting the complex immunological alterations in the host [12], [13]. Notably, hyperthermia is able to up-regulate the expression of heat shock protein in the tumor cells, and which are associated with antigen presentation, cross presentation and anti-tumor immunity [14]. All these data provide the rationale upon enhancing the anti-tumor effects of hyperthermia by combining with immune-modulatory agents.

Glycolipid, such as α-galactosylceramide (KRN7000, α-GalCer, a glycosphingolipid) has been shown to inhibit tumor growth and prolong survival in a few mouse tumor models through activation of innate and adaptive immune responses [15], [16]. Several clinical trials have also documented its safety and biological effects in patients with solid tumors through parenteral delivery [17], [18]. This compound itself is not a cytotoxic agent, but can be presented to NKT cells by CD1d [19], [20]. α-GalCer -activated NKT cells were reported to secrete large amounts of cytokines, including IFN-γ and IL-4, and thereby activating other effectors cells, such as NK cells, T cells, B cells, DCs and macrophages [16]. The administration of α-GalCer into animals could also induce the production of other cytokines, such as IL-2, IL-6, IL-12, and GM-CSF, which might also reinforce the immune system [21].

We hypothesized that hyperthermia is not only cytotoxic but also able to unveil the immunogenicity of tumor cells, directing the immune response over the microenvironment to both Th1/Th2 pathways. We proposed that the addition of α-GalCer may induce the activation of NKT cells as well as the maturation of DCs. Following treatment with α-GalCer, a strong cytokine cascade is subsequently elicited, thereby boosting the cross-talk between the innate and adaptive anti-tumor immune responses.

In this study, we utilized different murine ovarian cancer cells as models to test our hypotheses and to investigate the anti-tumor effect and underlying mechanisms of i.p. hyperthermia combined with α-GalCer in the treatment of ovarian cancer.

## Materials and Methods

### Mouse and Cell Line

C57BL/6 mice and BC3F1 (C57BL/6×C3H F1) mice were purchased from BioLASCO, Taiwan. The animals were cared under specific pathogen-free conditions. The mouse ovarian cancer cell line MOSEC-luc (C57BL/6 origin and engineered expression of firefly luciferase), ID8-luc (derived from MOSEC-luc with VEGF over-expression), and HM-1 (BC3F1 origin) were cultured in RPMI1640 medium (Gibco) supplemented with 10% FBS (Biological Industrial, Israel), 100 U/ml penicillin (Gibco) and 100 pg/ml streptomycin (Gibco) in a humidified atmosphere of 5% CO_2_/95% air at 37°C.

### Ethics Statement

All the experiments followed the guidelines with regards to experimental animal welfare and were permitted by IUAUC of Mackay Memorial Hospital (MMH-A-S-97030).

### Intra-peritoneal Hyperthermia and α-GalCer Treatment

To perform i.p. hyperthermia, exploratory laparotomy was carried out after the mice were anesthetized with Zoletil (VIRBAC, France) and Rompun (Bayer Vital GmbH, Leverkusen) first. The peritoneal cavity of every mouse was constantly filled with 45°C 1×PBS. Heated PBS was refilled to replace cooled PBS by the frequency of 20∼30 sec/cycle for a total of 10 minutes. In the combined treatment group, 2 µg of α-GalCer (Enzo Life science, USA) was added into the peritoneal cavity immediately after the completion of hyperthermia. The peritoneal cavity was also opened for 10 minutes in control group of mice. Mice were kept warm under heater lights until they recovered from anesthesia.

### Multiplex Serum Cytokine Detection

The venous blood harvested from the tail of each mouse was incubated at 37°C for 30 min then centrifuged at 1500×g for 10 min. The supernatant serum were transferred to another clean tube and stored at −20°C before analysis. The concentration of IL-2, IL-4, IL-5, IL-6, IL-13, INF-α, and TNF-α in serum of each mouse was detected by BD Cytometric Bead Array kit (BD bioscience) as described by the manufacturer.

### Primary Cell Isolation and NK/NKT Cell Detection

For isolating the white blood cells (WBC), the peripheral blood was collected from the tail vein of mice. The RBCs in blood were removed by ACK lysis buffer (Invitrogen) and the WBCs were then resuspended in RPMI 1640 before analysis. For isolating the cells within the peritoneal cavity, the mice were sacrificed by CO_2_ euthanasia. Cells within the peritoneal cavity were collected by peritoneal lavage with 3% BSA in cold 1× PBS. Cells recovered from lavage were washed and resuspended in RPMI 1640 medium. Cells were then stained by FITC-anti-CD3 and PE-anti-NK1.1 antibody as manufacture described (eBioscience). The populations of NK and NKT cells were detected by Flow cytometry. (Calibur, BD Bioscience). The results were analyzed by FACS express software.

### Detection of Cancer Cell Apoptosis

One million of ID8-luc cells were injected intra-peritoneally into each C57BL/6 mouse in the experimental group. Three days later, i.p. hyperthermia was given in the tumor-bearing mice as described previously. No intervention was performed in the control group of mice except opening/closing of the peritoneal cavity. One day later, mice were sacrificed by CO_2_ euthanasia. Cells inside the peritoneal cavity were lavaged and stained with Annexin V-APC and Propidium iodide (PI, BD bioscience), and then subjected to analysis by flow cytometry.

### Cytotoxicity of Peritoneal Effectors Cells

Twenty thousands of ID8-luc cells were seeded in each well of round bottom 96-well plates as target cells. Those plates were sealed with paraffin and put on the liquid surface of 42°C water bath and incubated for 20 min. The lavaged cells (from control group or α-GalCer-treated group of mice) as effectors cells were then added into the ID8-luc cell containing well with the effectors/target (E/T) ratio of 5∶1 and 10∶1, and cultured at 37°C overnight. After adding luciferin (Caliper), the amount of survived ID8-luc cells was presented as the intensity of chemoluminescence detected by the IVIS imaging system. (Xenogen).

### Dendritic Cell Phagocytosis Assay *in vitro*

Two micrograms of α-GalCer were i.p. injected into C57BL/6 mice and left overnight. The mice with and without α-GalCer treatment were sacrificed and the spleens were transferred to RPMI 1640 medium. The splenocytes were isolated by smashing the spleens on 100 µm cell strainer in 6 cm dish contain 5 ml of RPMI1640 medium. The suspended cells were washed and the RBCs were removed by ACK lysis buffer (Invitrogen). The DCs in those splenocytes were purified by anti-CD11c magnetic beads (MACS, Germany). For phagocytosis assay, ID8-luc cells were stained with 25 µM of CFSE (Sigma) and then were given heat treatment as previously described. Purified DCs from control group or α-GalCer-treated group of mice were then co-cultured with 1×10^5^ of either heated or non-heated CFSE-stained ID8-luc cells for 6 hr. DCs were labeling by staining with APC-anti-CD11c antibody (eBioscience). The percentage of CFSE-positive DCs was analyzed by flow cytometry with the gating of CD11c positive cells.

### Dendritic Cell Phagocytosis Assay *in vivo*

One million of CFSE-stained (25 µM) ID8-luc cells were i.p. injected into C57BL/6 mice. On the next days, all mice were divided into 4 groups and given i.p. hyperthermia and/or 2 µg of a-GalCer. Mice received laparotomy only (without drug treatment) was set as control group. After 24 hr, the intra-peritoneal cells were lavaged and stained with PE-anti-mouse CD11b and APC-anti-mouse CD11c antibodies (eBioscience). The percentage of CFSE-positive DCs was analyzed by flow cytometry by gating of CD11b^+^/CD11c^+^ positive cells.

### Tumor Specific CD8^+^ T Cell Immunoassay

One million of HM-1 cells were i.p. injected into B6C3F1 mice and 3×10^5^ of ID8-luc cells were injected intra-peritoneally into C57BL/6 mice. Three days later, the mice were anesthetized and treated i.p. with or without α-GalCer. After one or three weeks, the mice were sacrificed for isolating the splenocytes. Splenocytes (1×10^7^) were cultured with or without 1×10^5^ of the syngenetic cancer cells (those had been injected into the peritoneal cavity of the mice) with 1 µg of Golgi-plug (BD Pharmingen) in 24-well plates. On the next day, cells were stained with APC-conjugated monoclonal rat-anti-mouse CD8a (eBioscience) for 20 minutes, and fixed with Cytofix/Cytoperm kit (BD Pharmingen), followed by staining with FITC-conjugated rat anti-mouse interferon-γ (eBioscience) as manufacture described. The number of CD8/Interferon-γ positive cells was analyzed by flow cytometry.

### Non-invasive Tumor Growth Measurement

One million of MOSEC-luc cells were i.p. injected into C57BL/6 mice. After one week, i.p. hyperthermia was performed with or without α-GalCer treatment. Tumor growth of MOSEC-luc cells in mice was detected by non-invasive chemoluminescence imaging system (IVIS, Xenogen).

### Depletion of Lymphocyte Sub-populations

One million MOSEC-luc cells were i.p. injected into C57BL/6 mice. Five days later, mice were divided into 4 groups and received i.p. injection of either 100 µg anti-mouse CD4 (clone GK1.5), anti-mouse CD8 (clone TIB210) or rat IgG control (Millipore) antibodies at a 2-day interval. All the mice were given i.p. hyperthermia combined with 2 µg of α-GalCer one week after tumor inoculation. Tumor growth was measured by non-invasive IVIS imaging system as previously described.

### Statistics

All results were represented by mean ± SE (standard error) from at least two independent experiments. The comparisons between different data points were performed by using ANOVA test or a student’s T test. The survival was represented by Kaplan-Meier curve and compared by using long-rank analysis.

## Results

### Intra-peritoneal Hyperthermia Enhanced the Secretion of Pro-inflammatory Cytokines Induced by α-GalCer

**Figure 1:**
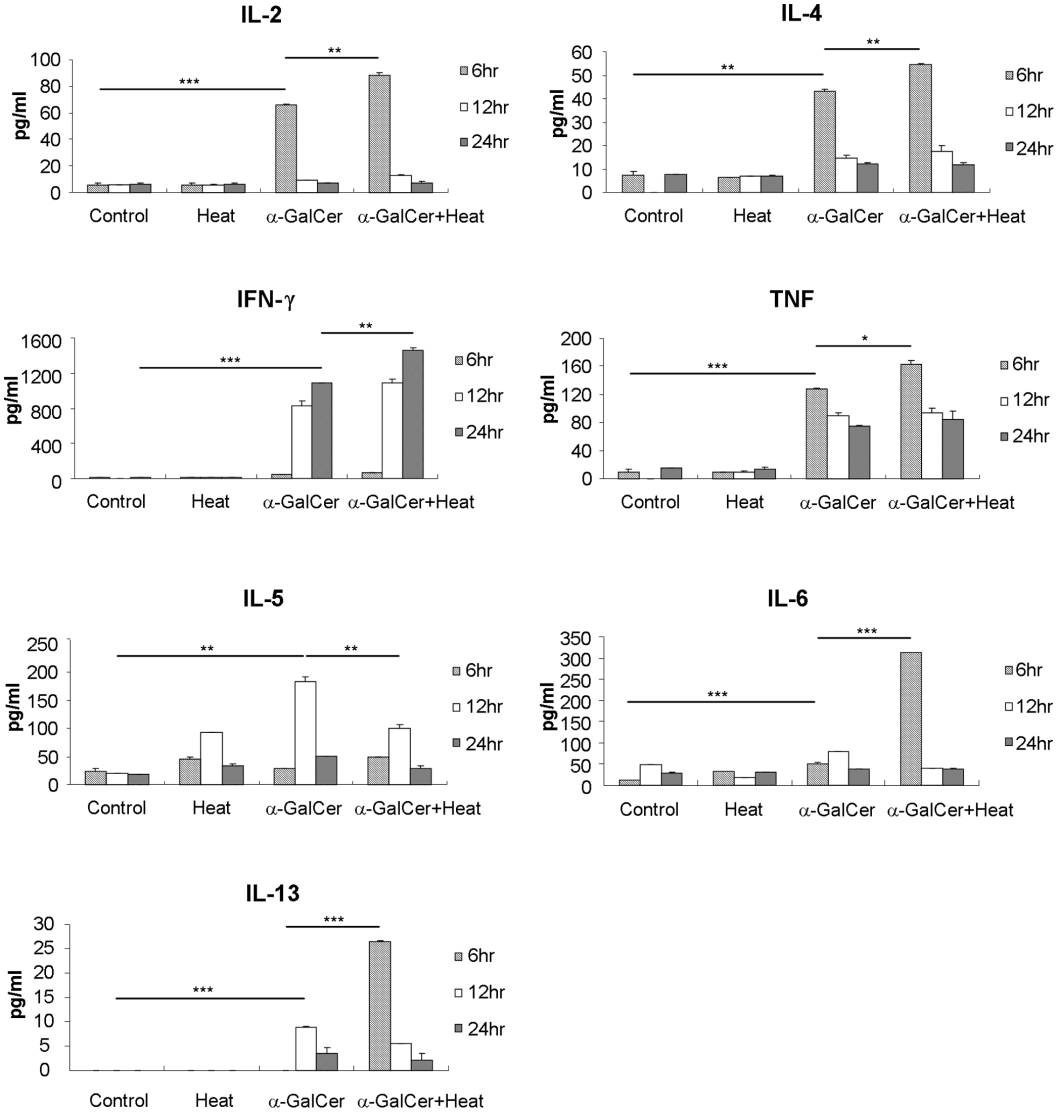
Th1/Th2 cytokine expression in serum of i.p. hyperthermia and/or α-GalCer treated mice. C57BL/6 mice were divided into 4 groups with 5 mice for each group and treated with i.p. hyperthermia only at 43°C for 10 min, i.p. added 2 µg of α-GalCer, and combining both. The control group mice were only performed exploratory laparotomy for 10 min. Mouse serum was collected at 6, 12, and 24 hr after treatment. IL-2, IL-4, IL-5, Interferon-γ, TNF-α, IL-6 and IL-13 concentrations of serum were detected by multiplex CBA kit. Some cytokine levels were increased by α-GalCer and peaked at 6 hr post-treatment (IL-2, p = 0.0003, α-GalCer alone *versus* control), (IL-4, p = 0.0012, α-GalCer alone *versus* control), (IL-6, p<0.0001, α-GalCer *versus* control), (TNF-α, p = 0.0007, α-GalCer *versus* control); Some peaked at 12 hr after treatment (IL-5, p = 0.0013, α-GalCer *versus* control) (IL-13, p<0.0001, α-GalCer *versus* control); one peaked at 24 hr after treatment (INF-γ, p<0.0001, α-GalCer *versus* control). Except for IL-5, the Th1/Th2 cytokine levels were higher in combined treatment group than α-GalCer alone group (IL-2, p = 0.0056; IL-4, p = 0.0041; TNF-α, p = 0.018; INF-γ, p = 0.0058). The peak level of IL-13 was reached earlier in the combined treatment group and its level was much higher than that of α-GalCer alone (p<0.0001). These results implied that α-GalCer treatment could trigger immune response toward Th1/Th2 route and hyperthermia could further enhance this effect. (# p<0.05; * p<0.01; ** p<0.001; *** p<0.0001).

The key anti-tumor effects of α-GalCer are thought to be through modulating the downstream immune cascades by stimulating Th1/Th2 cytokine production by NKT cells. Except for IL-5, i.p. hyperthermia further enhanced the secretion of these six cytokines initially elicited by i.p. α-GalCer treatment. We first examined whether the addition of i.p. hyperthermia is able to influence the expression of these cytokines stimulated by α-GalCer installation. Figure 1 shows that i.p. hyperthermia alone did not alter the levels of Th1/Th2 cytokines, whereas i.p. α-GalCer administration triggered the secretion of several cytokines, which reached their highest levels at different time points. Levels of IL-2 (p = 0.0003, α-GalCer *versus* control), IL-4 (p = 0.0012, α-GalCer *versus* control), IL-6 (p<0.0001, α-GalCer *versus* control), and tissue necrotic factor alpha (TNF-α) (p = 0.0007, α-GalCer *versus* control) peaked in 6 hours, while IL-5 and IL-13 peaked in 12 hours (p = 0.0013, α-GalCer *versus* control for IL-5; p<0.0001, α-GalCer *versus* control for IL-13). It took up to 24 hours for the IFN-γto achieve its peak level (p<0.0001, α-GalCer *versus* control). Except IL-5, i.p. hyperthermia further enhanced the secretion of these five cytokines initially elicited by i.p. α-GalCer treatment (p = 0.0056, for IL-2; p = 0.0041, for IL-4; p<0.0001 for IL-6; p = 0.0058, for IFN-γ and p = 0.018 for TNF-α). This mode of treatment also enhanced the secretion of IL-13 (p<0.0001, hyperthermia plus α-GalCer versus α-GalCer alone) which peaked earlier at 6 hr after treatment. These results indicated that α-GalCer might induce stronger anti-tumor immune response mainly through the enhancement of Th1/Th2 cytokines expression by combining with i.p. hyperthermia.

### α-GalCer Treatment Exhibited Synergistic Cytotoxic Effects Against Heated Ovarian Cancer Cells

**Figure 2:**
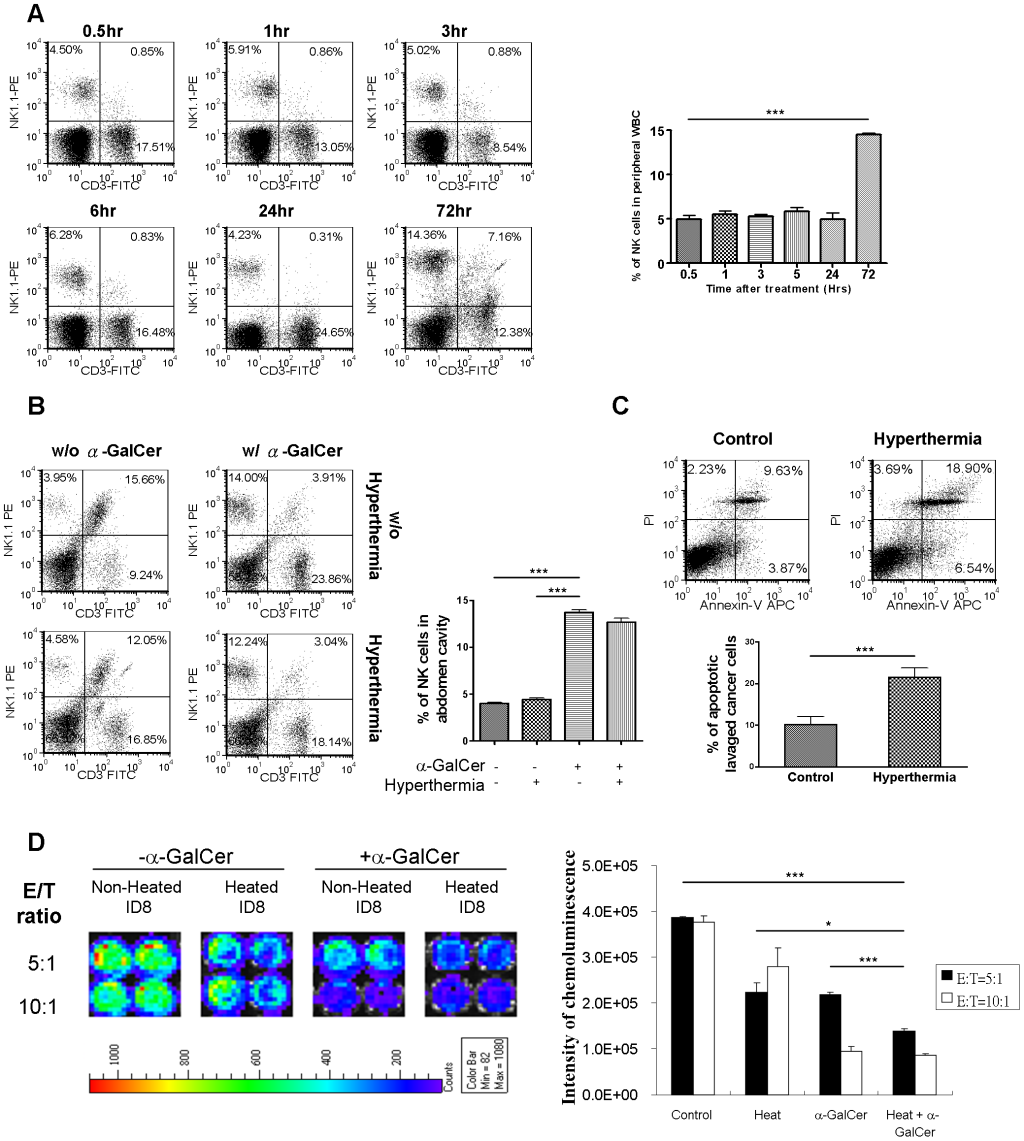
Synergistic anti-tumor effect of hyperthermia induced apoptosis of cancer cells and α-GalCer induced NK cells cytotoxicity in vitro. (A) 2 µg of α-GalCer was i.p injected into 5 C57BL/6 mice and the tail vein blood was collected at 0.5, 1, 3, 6, 24, and 72 hr after treatment. The proportion variation of NK cells in peripheral blood was analyzed by flow cytometry with fluorescent CD3 and NK1.1 antibody staining. The results demonstrated that α-GalCer significantly recruited the NK cell population in peripheral blood 72 hr after treatment. (p<0.001, 72 hr *versus* other time points) (B) C57BL/6 mice were divided into 4 groups and treated by intraperitoneal hyperthermia at 43°C, 2 µg of α-GalCer, or combination. The control group mice were only opened their abdomen for 10 minutes. Intra-peritoneal cells were harvested by peritoneal lavage 3 hours after treatment. Variation on the proportions of intra-peritoneal NK cells was analyzed by flow cytometry with fluorescent CD3 and NK1.1 antibody staining. The results implied i.p. α-GalCer administration increased local NK cell proportion within a few hours. Combination of hyperthermia did not compromise this effect. (p = 0.0007, α-GalCer *versus* control; p = 0.0009, α-GalCer *versus* hyperthermia; no significant different between α-GalCer alone and combined treatment) (C) ID8 tumor-bearing C57BL/6 mice were treated with intra-peritoneal hyperthermia at 43°C for 10 min. One day after treatment, intra-peritoneal cells were lavaged and stained with Annexin V and PI. The cancer cells were gated and the apoptotic index was analyzed by flow cytometry. The proportion of apoptotic cancer cells was increased after intra-peritoneal hyperthermia. (p = 0.0084, hyperthermia *versus* control) (D) 2×10^4^ of ID8-luc cells were seeded into 96-well round button plates and heated on 42°C water bath for 20 minutes. Different ratios of intra-peritoneal lavaged cells from mice i.p injected with or without α-GalCer were then added into the well as effectors cells and co-cultured overnight with heated or non-heated ID8-luc cells as target cells. Non-heated ID8-luc cells co-cultured with lavaged cells without treating α-GalCer were used as control. The cytotoxicity represented by the intensity of chemoluminence was the detected by IVIS imaging system. The results showed that co-culture of heated cancer cell with intra-peritoneal lavage cells from α-GalCer-treated mice induced highest cytotoxicity of cancer cell in vitro. (At E/T ratio = 5∶1, p<0.0001, heat plus α-GalCer *versus* control; p = 0.0162, heat plus α-GalCer *versus* heat; p = 0.0004, heat plus α-GalCer *versus* a-GalCer). (# p<0.05; ** p<0.001; *** p<0.0001).

It has been shown that the main anti-tumor effects induced by α-GalCer are through the release of certain cytokines, which augment the activities of NK or T cells. To investigate whether α-GalCer treatment has an impact on the activity of NK cells, we monitored the NK cell population at different time points after i.p. α-GalCer treatment. In peripheral blood, the NK cell population did not change significantly within first 24 hr. However, the percentage of NK cells dramatically increased 72 hr after the treatment (Figure 2A; p<0.001, 72 hr *versus* other time points). Inside the peritoneal cavity, NK cell population increased in 3 hours after α-GalCer administration, and addition of i.p. hyperthermia did not affect the increments (Figure 2B).

Hyperthermia has been reported to cause death of cancer cells. In ID8 tumor-bearing mice, more apoptotic ID8 cells (i.p.) was found in mice treated by hyperthermia than those in the control mice (Figure 2C; p = 0.0084, hyperthermia *versus* control).

In the *ex-vivo* co-culture, it was also found that heated ID8 cells were more susceptible to peritoneal lavaged cells obtained from α-GalCer-treated mice (Figure 2D; p = 0.0004, heated *versus* non-heated ID8 cells at E/T ratio 5∶1). These results implied that i.p. administration of α-GalCer might recruit more NK cells, leading to greater cancer cell killing, and hyperthermia induces an additional cytotoxic effect to cancer cells.

### Treatment Combining i.p. Hyperthermia with α-GalCer Generated a Synergistic Effect on the DCs’ Phagocytic Activities *in vitro and in vivo*

**Figure 3:**
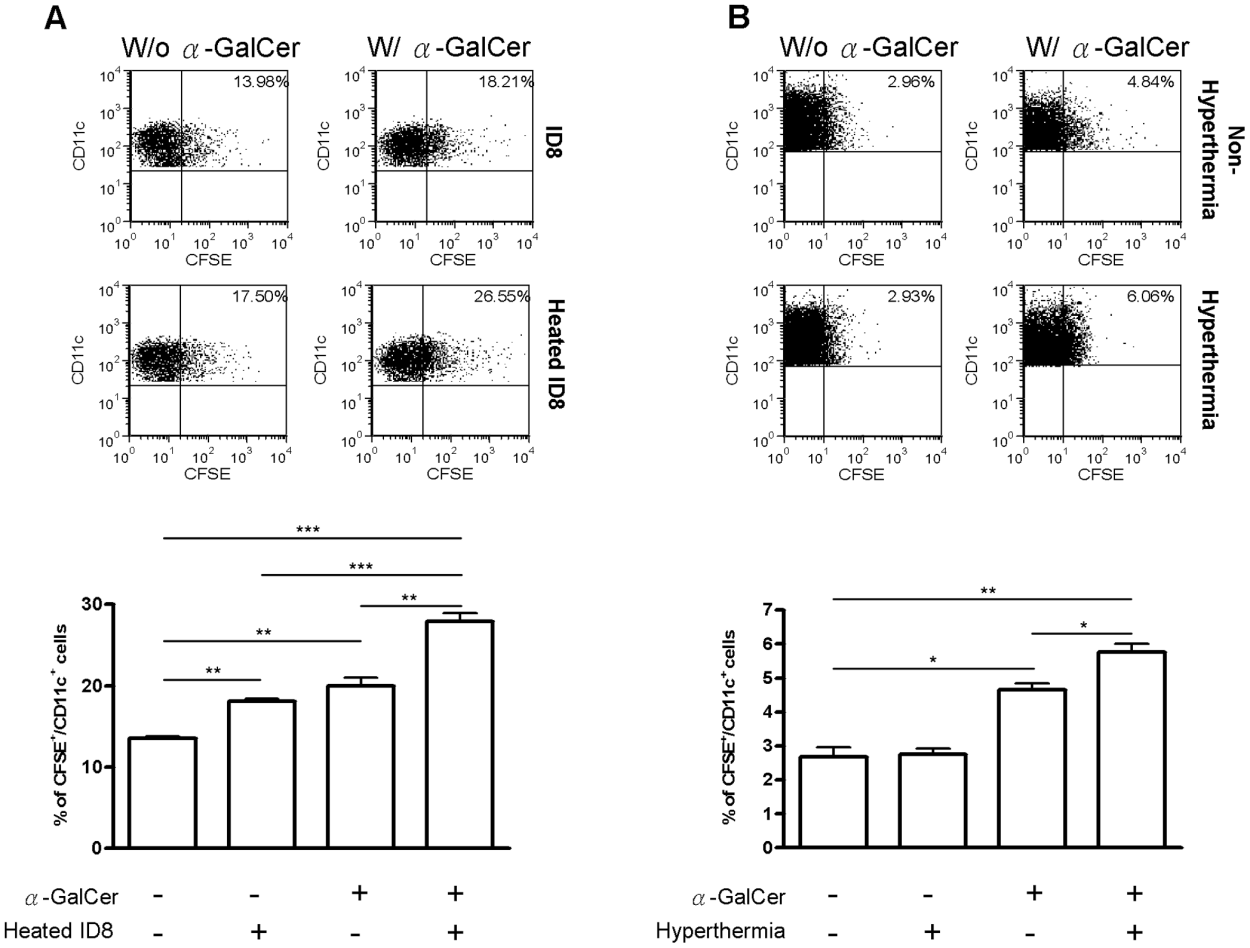
α-GalCer stimulation and heat on cancer cell synergistically enhanced the phagocytosis activity of DCs both *in vivo and in vitro.* (A) C57BL/6 mice were first i.p. injected with 2 µg of α-GalCer. After 18 hr, mice were sacrificed and the DCs were purified from spleens by anti-CD11c magnetic beads. For dendritic cell phagocytosis assay, ID8 cells were first stained with CFSE and heated on 42°C water bath for 20 minutes. The DCs from α-GalCer stimulated (or not-stimulated) mice were co-cultured with heated or non-heated ID8 cells for 6 hr at the ratio of 5∶1. The proportion of DCs with phagocytic activity was represented as CFSE positive in gated CD11c expressing cells analyzed by flow cytometry. The results showed that both *in vitro* heat treatment on cancer cells and *in vivo* stimulation of DC by α-GalCer could enhance the phagocytic activity of DCs (p = 0.005, heat *versus* control; p = 0.0037, α-GalCer *versus* control). The synergistic effect emerged in the combined treatment (p = 0.0002, combined treatment *versus* control; p = 0.0008 combined treatment *versus* heat; p = 0.005, combined treatment *versus* α-GalCer). (B) C57BL/6 mice were i.p. injected with 1×10^6^ CFSE-stained ID8 cells. On the next day, the mice were divided into four groups with treatment by i.p. hyperthermia and/or α-GalCer as previously described. One day later, intra-peritoneal cells were harvested through i.p. lavage and the proportion of CFSE-positive CD11b^+^/CD11c^+^ cells indicating phagocytic activities of DCs were analyzed by flow cytometry. It is shown that α-GalCer treatment could enhance the phagocytosis of DC *in vivo* (p = 0.0289, α-GalCer *versus* control). This effect was further enhanced by additional hyperthermia (p = 0.038, combined treatment *versus* α-GalCer). (# p<0.05; * p<0.01; ** p<0.001).

One of the anti-tumor activities of NKT cells involves the activation of DCs. The main function of DCs in anti-tumor immunity is to engulf the tumor cells and present tumor antigens to the relevant effectors cells, such as cytotoxic T cells. We further investigated the resultant effects of α-GalCer treatment with hyperthermia on phagocytic activities of DCs. Based on co-culture of cancer cells with DCs isolated from spleen, it is found that heated cancer cells were more susceptible to DCs’ phagocytosis (Figure 3A; p = 0.005, hyperthermia *versus* control). Moreover, DCs isolated from α-GalCer-treated mice exhibited significantly higher activities of phagocytosis (p = 0.0037, α-GalCer *versus* control). Putting together, co-culture of the heated cancer cells with DCs obtained from α-GalCer-treated mice demonstrated much more phagocytosis (p = 0.0002, combined treatment *versus* control; p = 0.0008, combined treatment *versus* hyperthermia; p = 0.005, combined treatment *versus* α-GalCer). These results showed that α-GalCer treatment plus hyperthermia leading to a synergistic effect on DCs’ phagocytosis *in vitro.* Given that the combined treatment was administered to target intra-peritoneal cancer cells, we analyzed the phagocytic activities of intra-peritoneal DCs obtained through peritoneal lavage. By counting the number of CFSE-stained CD11b^+^/CD11c^+^ DCs, it is noted that α-GalCer enhanced the phagocytic activities of i.p. DCs (p = 0.0289, α-GalCer *versus* Control) and hyperthermia plus α-GalCer further enhanced this effect (p = .0.038, combined *versus* α-GalCer alone). These results clearly showed that α-GalCer treatment plus hyperthermia also leads to a synergistic effect on DCs’ phagocytosis inside the peritoneal cavity.

### Combination of Intra-peritoneal Hyperthermia and α-GalCer Elicited a Tumor-Specific Cytotoxic CD8^+^ T Cell Immune Response

**Figure 4:**
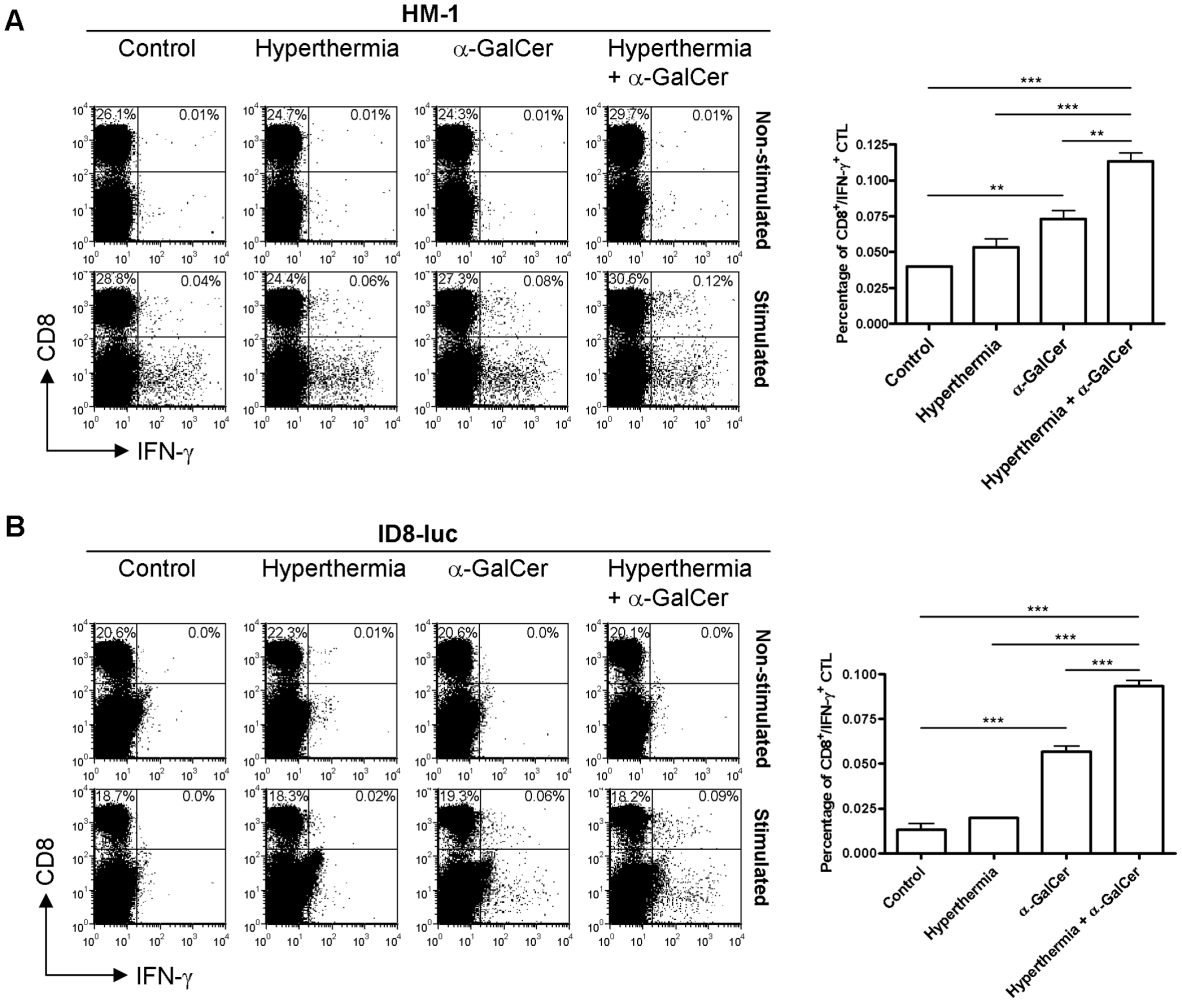
Hyperthermia combined α-GalCer stimulation activated tumor-specific cytotoxicity T cell response in tumor-bearing mice upon different mouse ovarian cancer models. (A) 1×10^6^ of HM-1 cells were i.p injected into B6C3F1 mice and (B) 3×10^5^ of ID8-luc cells were i.p. injected into C57BL/6 mice. Mice were divided into four groups (untreated control, hyperthermia only, α-GalCer alone and combined treatment with hyperthermia plus α-GalCer) with five mice per group and treated as described above. After 7 days, the splenocytes were isolated and stimulated overnight with ID8 cells or HM-1 cells. The splenocytes from each group without re-stimulated with ID8 cells or HM-1 cells were also cultured overnight as control. The percentage of cancer specific CTL represented as CD8^+^/IFN-γ^+^ were detected by flow cytometry by gating of 2×10^5^ lymphocytes. It is shown that, 7 days after treatment, i.p. α-GalCer treatment slightly increased the number of tumor specific CTL (p = 0.0033, control *versus* α-GalCer for HM-1; p<0.0001, α-GalCer *versus* control for ID8). Hyperthermia plus α-GalCer exhibited strongest effect in activating specific CTL (p = 0.0026, combined treatment *versus* α-GalCer for HM-1; p<0.0001, combined treatment *versus* α-GalCer for ID8). (* p<0.01; *** p<0.001).

Since α-GalCer treatment can enhance the phagocytic activities of DCs and hyperthermia can further strengthen this effect, the next question would be whether combination of i.p. hyperthermia and α-GalCer treatment could induce tumor-specific cytotoxic T cell response. To assess the presence of cellular immune response against cancer cells, two different mouse ovarian cancer cells, HM-1 and ID8-luc, were used as immuno-competent tumor models. Seven days after treatment, treatment of HM-1-bearing mice with α-GalCer alone merely increase a small number of tumor-specific CD8^+^ T cells within the splenocytes (p = 0.0033, α-GalCer alone *versus* control). However, we found a significant increase in the number of tumor-specific CD8^+^ T cells in HM-1-bearing mice treated with i.p. hyperthermia plus α-GalCer (p<0.0001, combined treatment *versus* control; p<0.0001, combined treatment *versus* hyperthermia; p = 0.0026, combined treatment *versus* α-GalCer alone, Figure 4A). In ID8-luc model, tumor- specific CD8^+^ T cell immune response could be detected in the group treated with α-GalCer alone (p<0.0001, α-GalCer alone *versus* control). However, this effect was found to be more significant in the group treated with combinational therapy (p<0.0001, combined treatment *versus* other groups). The enhancement of tumor specific CTL response was diminished by 21 days after treatment (data not show). This may be due to overgrowth of tumor cells which restrained the anti-tumor immune response induced by only one-time treatment. Theoretically it would be much effective if hyperthermia can be performed repeatedly, or the tumor growth might eventually overcome the anti-tumor immune response in this rapid-growing tumor model. These results demonstrated that i.p. hyperthermia can further enhance the cytotoxic tumor-specific CD8^+^ T cell immune response induced by α-GalCer treatment.

### Intra-peritoneal Hyperthermia Enhanced the Therapeutic Effects of α-GalCer *in vivo*

**Figure 5:**
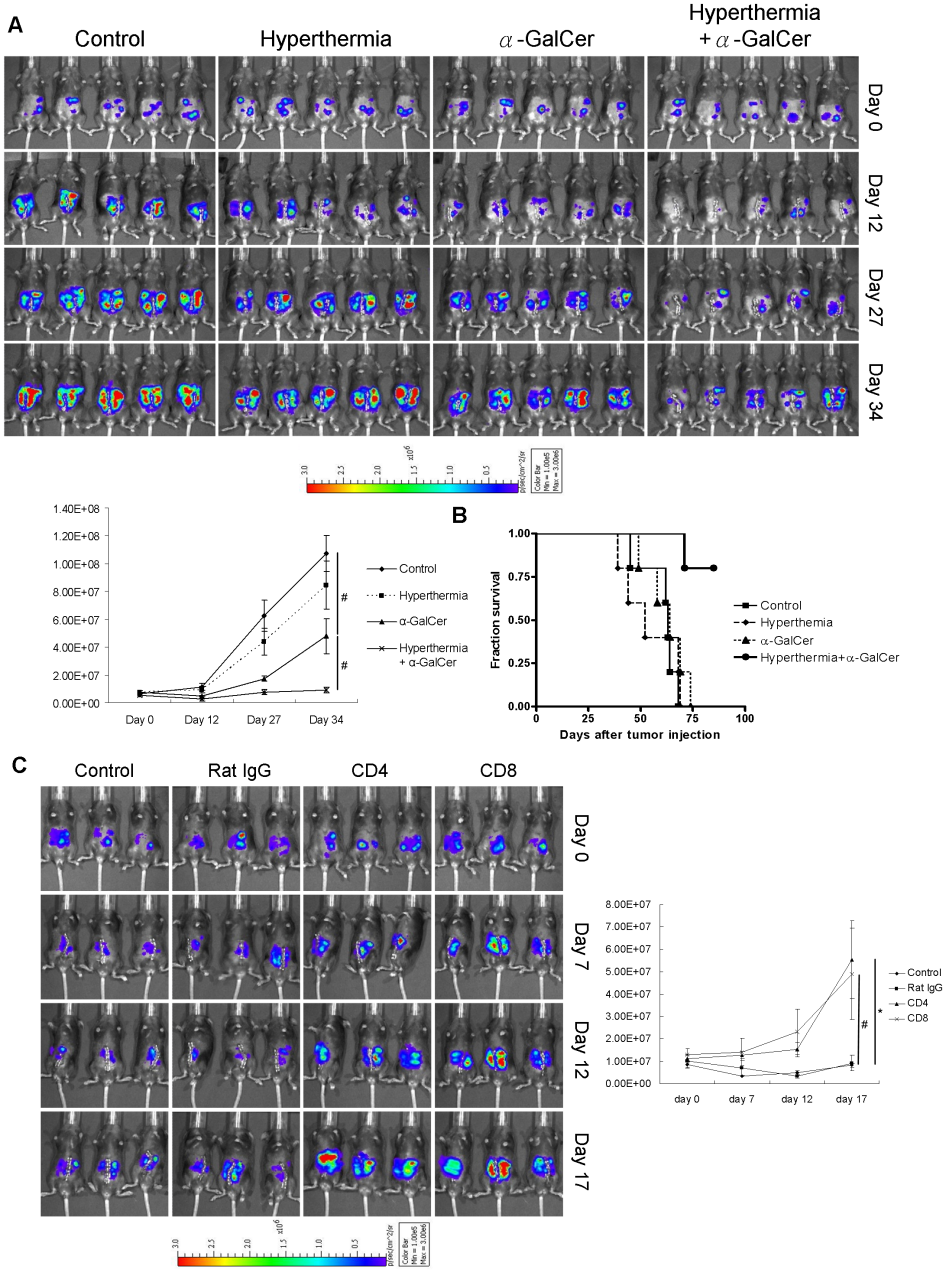
Intra-peritoneal hyperthermia enhanced the anti-tumor effect of α-GalCer in mouse ovarian cancer model. (A) One million of MOSEC-luc cells were i.p. injected into C57BL/6 mice. Seven days later, the mice were divided into 4 groups and treated as described previously. Tumor growth was recorded and represented by the intensities of chemo-luminescence detected by IVIS imaging system. The results showed that α-GalCer could inhibit the tumor growth and the combined treatment exerted even stronger inhibition. (B) Survival curve on each group revealed that there is no significant difference in survival of control, hyperthermia alone and α-GalCer treated groups. However, combinational treatment could clearly extend the survival of the treated mice. (C) One million of MOSEC-luc cells were i.p. injected into C57BL/6 mice. Seven days later, the mice were divided into 4 groups and received i.p. injection of either 100 µg of rat IgG, rat anti-mouse CD4 or rat anti-mouse CD8 neutralizing antibody every 2 days. Mice without injection of neutralizing antibodies were set as control group. Intra-peritoneal hyperthermia plus α-GalCer treatment was given to all mice on day 7. Tumor growth was recorded and represented by the intensity of chemo-luminescence detected by non-invasive IVIS imaging system. The results showed that administration of mouse CD4 and CD8 neutralizing antibodies abrogated the anti-tumor effect, which implied both CD4^+^ and CD8^+^ T cell contribute to the anti-tumor effect. (# p<0.05; *p<0.01).

It is evident that i.p. hyperthermia enhanced both innate and adaptive anti-tumor immune responses induced by α-GalCer treatment. We validated whether this combinational treatment could result in better therapeutic effect *in vivo*. In MOSEC-luc tumor-bearing C57BL/6 mice, i.p. hyperthermia plus α-GalCer exhibited stronger inhibition of tumor growth (Figure 5A; p<0.05, combined treatment *versus* α-GalCer alone). And this treatment also prolonged the survival time of tumor-bearing mice (Figure 5B; p = 0.0018, combined treatment *versus* control). However, no significant anti-tumor effect could be demonstrated in the group treated with i.p. hyperthermia alone. These results implied that i.p. hyperthermia, though did not elicit significant anti-tumor effect, do enhance tumor growth inhibition induced by α-GalCer treatment, leading to a better survival in mice.

To further confirm that T cell mediated immune responses contribute to the anti-tumor effects of hyperthermia plus α-GalCer treatment, CD4+ and CD8+ T cells were depleted by giving neutralizing antibodies. The administration of both anti-mouse CD4 and anti-mouse CD8 neutralizing antibodies abrogated the anti-tumor effect of the combined treatment (p = 0.034, anti-CD4 *versus* control; p = 0.0245, anti-CD8 *versus* control). There is no significant difference in the tumor growth between control group and group administered with rat IgG. These results demonstrated that CD4^+^ and CD8^+^ T cells contribute to the anti-tumor effect of hyperthermia plus α-GalCer treatment.

## Discussion

It has been always believed that one of the approaches to successful treatment of ovarian cancer is to prevent the cancer cells from their escape from immune surveillance or to boost anti-tumor immune response of the host. α-GalCer has long been reported to have potential anti-tumor effect through the activation of host anti-tumor immunity. However, it has also been reported that organisms treated with α-GalCer developed anergy of NKT cells and the associated reaction might take a long period of time to be reactivated after stimulation [22]. Though the anergy of NKT cells might potentially skew Th2 cytokine production [23], [24], this limitation might be one of the causes of its failure in clinical application for cancer treatment. Thus, in order to make the best of this compound in cancer treatment, several ways had been explored to enhance and extend the initial anti-tumor effects of α-GalCer. The reports have utilized α-GalCer-loaded tumor or DCs to reinforce iNKT-mediated anti-tumor therapy [25], [26]. In our study, hyperthermia plus α-GalCer enhanced the expression of a variety of pro-inflammatory cytokines stimulated by α-GalCer, and thus drive the subsequent anti-tumor immune cascades.

The anti-tumor effects of α-GalCer are commenced by innate immune response of NK cells and then adaptive cellular immune response presented by DCs through the pro-inflammatory cytokines. α-GalCer recruits NK cells locally in short time and increases systemic NK cells eventually. We found that i.p. hyperthermia did not affect local NK cell recruitment. Peritoneal cells from mice treated with α-GalCer exhibited stronger cytotoxic activity against cancer cells, and heat to the cancer cells additionally strengthen this effect. This result probably comes from the simultaneous occurrence of cancer cell apoptosis induced by i.p. hyperthermia and the NK activities induced by α-GalCer. On the other hand, it seems that hyperthermia can strengthen the phagocytic abilities of DCs in the α-GalCer-treated mice, leading to more tumor antigen presentation to cellular immune system and trigger pronounced adaptive immune response. Not only on immunogenic cancer cells, the combination of i.p. hyperthermia and α-GalCer treatment also increased the tumor specific CD8^+^ cytotoxic T cell response on relatively poor immunogenic cells. The enhancement of adaptive immune response significantly potentiated the anti-tumor effects of α-GalCer and prolonged the survival of tumor bearing mice. This combined modality might be applied to treat drug-resistant tumor as an alternative adjuvant.

It is not yet clear why the combination of i.p. hyperthermia with α-GalCer treatment can induce stronger CD8^+^ cytotoxic T cell response. Hyperthermia causes more death of cancer cells, while does not compromise the immune systems as chemotherapies do [27], [28]. Apoptosis of cancer cells has been reported to induce adaptive anti-tumor immune response through expression of some molecules, such as surface calreticulin, Hsp70, Hsp90 and soluble HMGB1 (high-mobility group box 1 protein). These molecules can trigger a “eat-me” signal that induces the engulfment of these dying cells by DCs [14], [29], [30]. The temperature set for i.p. hyperthermia is so-called “mild hyperthermia” that may cause cancer cell apoptosis, instead of necrosis that is usually caused by high temperature. This treatment might cause more dying cells which raise “eat-me” signals, leading to the enhancement of phagocytosis process of DCs and then the presentation of tumor antigens to lymphocytes. It has also been reported that tumor regression after heating does not occur in immunosuppressed hosts [31]. In our results, this combinational treatment increased CD8^+^ cytotoxic T cell immune response in different ovarian cancer mouse models. α-GalCer enhances the DCs engulfment of those dying cells and then the antigen presentation to adaptive immune system. This is probably the reason why the tumor specific CD8^+^ cytotoxic T cell immune response was strengthened by combinational treatment.

In conclusion, i.p. hyperthermia enhances the innate immune response induced by α-GalCer, including pro-inflammatory cytokine production, NK cell activity and phagocytic activity of DCs. These effects further activate tumor specific cytotoxic T cell immune response and strengthen the anti-tumor effects induced by α-GalCer treatment. On the other hand, unlike conventional hyperthermic intra-peritoneal chemotherapy (HIPEC), combination of hyperthermia and α-GalCer inherits fewer side effects to patients and is safer to surgical personnel. Although this approach warrants more investigations before being applied clinically, it might lead to the development of a better therapeutic strategy of i.p. treatment for ovarian cancer.

## Supporting Information

Figure S1 Proportion of NK cells, DCs, and T cell subpopulation in the spleen and peritoneal cavity of different groups of mice after treatment. One million of MOSEC-luc cells were i.p. injected into C57BL/6 mice. Seven days later, all mice were divided into 4 four groups (5 mice for each group) and given intra-peritoneal hyperthermia with or without α-GalCer treatment as described previously. The group treated with laparotomy only was set as control. Thirty four days after treatment, the mice were sacrificed and the proportion of NK cells, DCs, and T cell sub-population in the splenocytes (A) and in the peritoneal cavity (B) were analyzed by flow cytometry. There was not significant difference in the proportion of NK cells, DCs, and T cell subpopulation in both spleen and peritoneal cavity long after different treatment. It implied that these therapeutic modalities did not affect the distribution of NK cells, DCs, and T cell subpopulation long after treatment. (n.s., non-significant).
(TIF)
