# Supplementary figures and images for: Intra-Peritoneal Hyperthermia Combining α-Galactosylceramide in the Treatment of Ovarian Cancer
Source: PLoS One. 2013 Jul 23;8(7):e69336. doi: 10.1371/journal.pone.0069336 (PMC3720534; doi:10.1371/journal.pone.0069336)

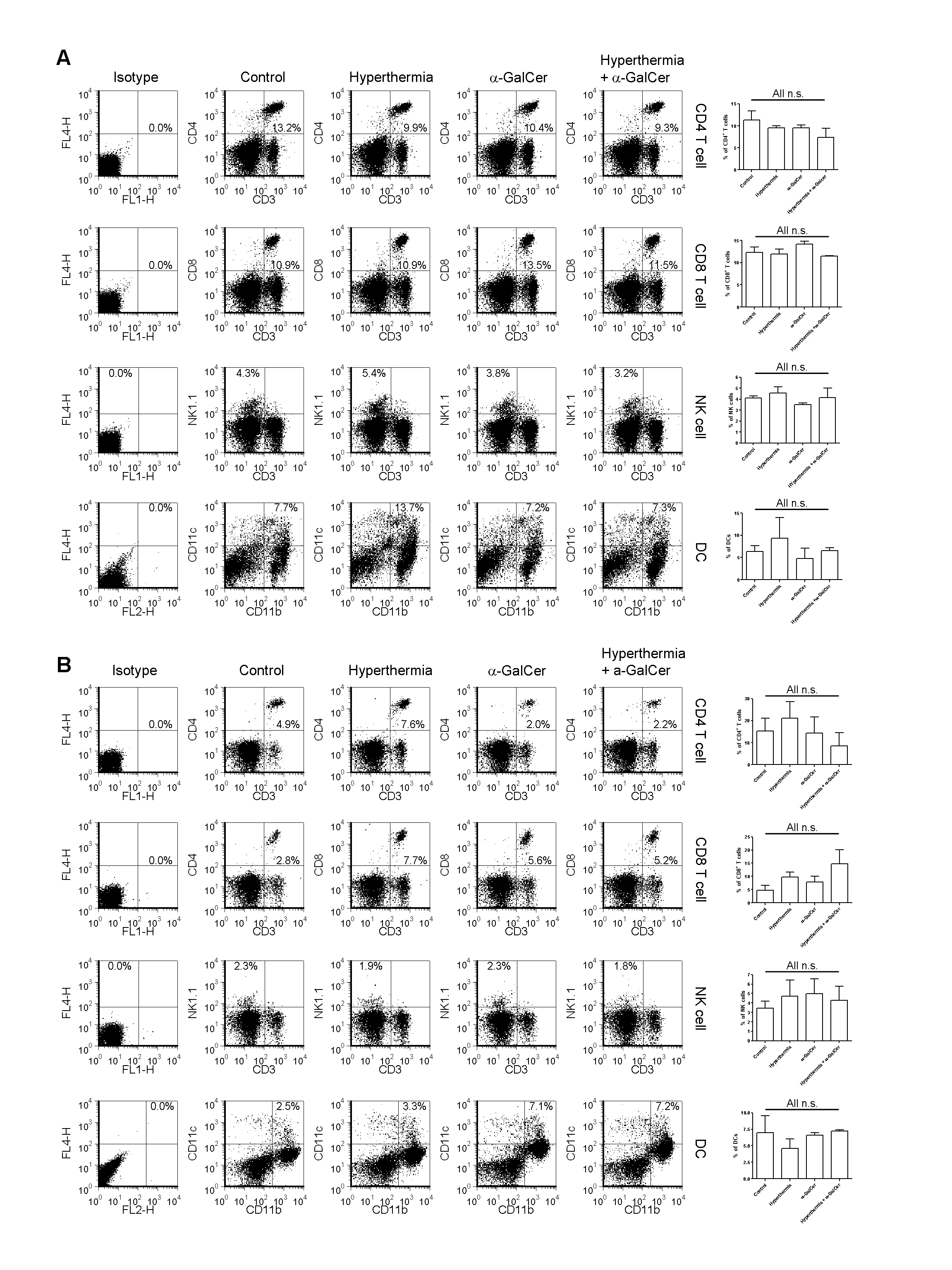

Supplement: Figure S1 — Proportion of NK cells, DCs, and T cell subpopulation in the spleen and peritoneal cavity of different groups of mice after treatment. One million of MOSEC-luc cells were i.p. injected into C57BL/6 mice. Seven days later, all mice were divided into 4 four groups (5 mice for each group) and given intra-peritoneal hyperthermia with or without α-GalCer treatment as described previously. The group treated with laparotomy only was set as control. Thirty four days after treatment, the mice were sacrificed and the proportion of NK cells, DCs, and T cell sub-population in the splenocytes (A) and in the peritoneal cavity (B) were analyzed by flow cytometry. There was not significant difference in the proportion of NK cells, DCs, and T cell subpopulation in both spleen and peritoneal cavity long after different treatment. It implied that these therapeutic modalities did not affect the distribution of NK cells, DCs, and T cell subpopulation long after treatment. (n.s., non-significant). (TIF) [file pone.0069336.s001.tif]
